# Editorial: Internet Gaming Disorder: A Pathway Towards Assessment Consensus

**DOI:** 10.3389/fpsyg.2019.01822

**Published:** 2019-08-06

**Authors:** Vasileios Stavropoulos, Rapson Gomez, Frosso Motti-Stefanidi

**Affiliations:** ^1^School of Psychology, Cairnmillar Institute, Melbourne, VIC, Australia; ^2^Discipline of Psychology, National and Kapodistrian University of Athens, Athens, Greece; ^3^Discipline of Psychology, Federation University, Ballarat, VIC, Australia

**Keywords:** internet gaming, disorder, measurement, assessment, scales

The use of video-games, either online or offline, has significantly increased, and almost homogeneously, around the globe over the last decades (Anderson et al., 2017). The majority of gamers have benefited from this rapid growth, which has a mostly positive effect on the cognitive, emotional, and social domains, as well as in their general well-being and everyday functioning (Jones et al., 2014).

In this context, the expansion of the video-gaming market has inevitably generated significant profits for the gaming producing industry and even employment opportunities for high-skilled and/or experienced gamers (Zhang and Fung, 2014). Nevertheless, this undoubtedly significant progress in the field of video-gaming has been accompanied by an equally significant downside for a considerable minority of gamers, who appear to have been overly-consumed by their gaming involvement (Stavropoulos et al., 2019a). Social withdrawal, reduced academic and work performance, as well as higher risk for a range of psychopathological behaviors including Depression, Anxiety, Attention Deficit and Hyperactivity and even Antisocial manifestations have been linked to excessive gaming (Stavropoulos et al., 2019b).

These negative outcomes have led to the adoption of various terms and definitions aiming to conceptualize gaming abuse as a modern psychopathological concern (Kuss et al., 2017). Despite the heterogeneity in the terms used to describe the phenomenon, the need to acknowledge the existence of a distinct clinical entity related to disordered gaming became apparent (Petry et al., 2014). Subsequently, the need to accurately define the fine line between disordered and adaptive gaming, so as to avoid pathologizing recreational gaming-engagement, has become pressing (Kardefelt-Winther et al., 2017). In this line, the development of clear diagnostic boundaries between disordered gaming and other clinical entities, that will allow differential diagnosis, emerged as an important goal (Scerri et al., 2019).

The American Psychiatric Association in the 5th edition of the Diagnostic and Statistical Manual for Mental Disorder (DSM-5; American Psychiatric Association, 2013) introduced a provisional classification of Internet Gaming Disorder (IGD), and invited scientists to conduct more research on the topic. Furthermore, the World Health Organization in the 11th edition of the International Classification of Diseases (ICD-11; World Health Organization, 2019) recently added the diagnosis of Gaming Disorder (GD) in its classification system. These developments have significantly contributed to addressing these needs.

However, the relative agreement in the definition of the construct that has been achieved, which constitutes a necessary requirement for the valid and reliable assessment of disordered gaming behaviors, is not sufficient (Stavropoulos et al., 2019a,b,c). Adequate psychometric properties of the scales utilized, to assess the disordered gaming classifications officially defined, are required for the accurate estimation and the cross-countries comparability of the syndrome's prevalence and incidence rates (Gomez et al., 2018). Hence, the development of valid diagnostic measurements that can inform disordered gaming clinical and prevention practices/protocols across different populations is necessary (Stavropoulos et al., 2018). Interestingly, and despite the ongoing, often chaotic and confusing, debate around the disordered gaming construct, the need of robust measures to psychometrically measure it has been stressed (Stavropoulos et al., 2018). In that line, significant progress has been made in regards to defining, understanding and confirming: (a) The dimensional-structure of the behavior; (b) How different criteria and scores translate (metric and scalar invariance) across populations; (c) Differential diagnostic criterion functioning (through the use of item response theory) and; (d) the psychometric stability of disordered gaming measurement over time (Kuss et al., 2017; De Palo et al., 2018; Gomez et al., 2018; Pontes et al., 2019; Stavropoulos et al., 2019c).

In this context, the goal of the present special topic-issue is to contribute to the ongoing discussions concerning this phenomenon. The studies included utilized culturally and developmentally diverse, normative samples from Iran (Lin et al.), the USA (Sprong et al.), Norway (Finserås et al.), Italy (Vegni et al.), Greece, Cyprus, and Australia (Hu et al.). Online gender specific (Lopez-Fernandez et al.) and face to face data collection procedures (Sprong et al.) were applied, in conjunction with a number of different models and analytical methodologies ranging from Confirmatory Factor Analysis (CFA; Hu et al.), Mokken analysis (Finserås et al.), Rash analysis (Lin et al.), Classical Test Theory (Hu et al.), Complex Regressions (Lopez-Fernandez et al.), and the PRISMA guidelines for systematic literature reviews (Costa and Kuss). Disordered gaming scales were assessed comparatively (Lin et al.), across genders (Lopez-Fernandez et al.), while the differential functioning of disordered gaming criteria was examined (Lin et al.; Sprong et al.; Finserås et al.).

The findings of this special topic contribute to the extant literature by shedding light to much debated, yet important, aspects of the assessment and measurement of disordered gaming behaviors. Indicatively: (a) the inclusion of gaming motivation as an inherent part of the assessment of disordered gaming behaviors has been supported by Sprong et al.; (b) Cultural values of independence, competitiveness and hierarchy (in the context of vertical-individualism) have been suggested to confound the assessment of the experienced level of absorbance by the gaming activity (online Flow; Hu et al.); (c) the need of particular emphasis on female gamers and their specialized assessment was emphasized (Lopez-Fernandez et al.); (d) a considerable delay in the employment of consistent measurements/ assessment in studies of clinically diagnosed disordered gamers was illustrated (Costa and Kuss); and (e) analogies with the emergence of gambling behaviors among younger individuals became clearer in the context of the broader literature (Vegni et al.).

However, challenges in the field of disordered gaming assessment still remain. Scholars continue to disagree about the nature of the behavior (Kardefelt-Winther et al., 2017), different instruments hindering international comparability are still employed (Costa and Kuss), while the number of measurement invariance studies, targeting in particular issues of scalar invariance (whether the same scores indicate the same severity) across populations of different genders, cultures, and developmental stages (although increasing) are rare (Stavropoulos et al., 2018, 2019c). The application of modern psychometric methodologies such as network analysis, that would illustrate the nature of the associations between the different criteria, is absent; while there is concurrently a dearth of Item Response Theory invariance studies to better highlight the potential different diagnostic functioning of certain criteria across different populations (Gomez et al., 2018). In this context, our conclusion is two-fold. First, that independently of the establishment or not of consensus around the definition of disordered gaming as a construct (Petry et al., 2014), *assessment and measurement discipline* in regards to the officially introduced definitions of DSM-5 (American Psychiatric Association, 2013) and ICD-11 (World Health Organization, 2019) is essential. Such discipline is expected to ensure higher prevalence and clinical diagnostic accuracy in relation to disordered gaming behaviors presenting globally and to significantly improve their efficient diagnosis. Second, the significant psychometric and cross-cultural progress in the field, especially after the introduction of the IGD definition (American Psychiatric Association, 2013) and the global expansion of the IGD associated scales is imperative to be acknowledged and utilized.

## Ethics Statement

All procedures performed in the study involving human participants were in accordance with the ethical standards of the institutional and/or national research committee and with the 1964 Helsinki declaration and its later amendments or comparable ethical standards. This article does not contain any studies with animals performed by any of the authors. Informed consent was obtained from all individual participants included in the study.

## Author Contributions

VS and RG contributed to the literature review, the structure, and sequence of theoretical arguments. FM-S contributed to the theoretical consolidation of the current work, revised, and edited the final manuscript.

### Conflict of Interest Statement

The authors declare that the research was conducted in the absence of any commercial or financial relationships that could be construed as a potential conflict of interest.
